# Effect of weight loss on pregnancy outcomes, neuronal-reproductive-metabolic hormones and gene expression profiles in granulosa cells in obese infertile PCOS patients undergoing IVF-ET

**DOI:** 10.3389/fendo.2022.954428

**Published:** 2022-09-30

**Authors:** Limin Wu, Qunying Fang, Mengli Wang, Yurui Wang, Xinyi Zhu, Zhaohui Fang, Fangting Lu, Bo Xu, Rentao Jin, Hui Han, Xianhong Tong

**Affiliations:** ^1^ Reproductive and genetic branch, The First Affiliated Hospital of USTC, Division of Life Sciences and Medicine, University of Science and Technology of China, Hefei, China; ^2^ Graduate school, Anhui University of Traditional Chinese Medicine, Hefei, China; ^3^ Endocrine Department, The First Affiliated Hospital, Anhui University of Traditional Chinese Medicine, Hefei, China; ^4^ Neurology Department, The First Affiliated Hospital, Anhui University of Traditional Chinese Medicine, Hefei, China

**Keywords:** Polycystic ovary syndrome, weight loss, pregnancy outcome, neuronal-reproductive-metabolic hormones, granulosa cells, gene expression, obesity

## Abstract

**Objective:**

To investigate the effect of weight loss on pregnancy outcomes, PCOS related neuronal-reproductive-metabolic hormones and ovarian granulosa cell gene expression profiles in obese PCOS infertile patients undergoing *in vitro* fertilization-embryo transfer (IVF-ET).

**Methods:**

75 patients undergoing IVF-ET due to tubal factors alone collected as the control group (group A), and 352 patients with obese PCOS infertility were divided into four groups according to the amount of weight loss before IVF: 0 kg (group B), 1-5 kg (group C), 5-10 kg (group D), and >10 kg (group E). Six cases of ovarian granulosa cells were collected randomly with the random number table method in each group for detecting mRNA profiling. Pathway networks and biological functions of the differentially expressed genes were analyzed. Validation by RT-PCR was performed.

**Results:**

(1) The levels of luteinizing hormone(LH), testosterone(T) and homeostasis model assessment insulin resistance(HOMA-IR) in group E were significantly lower than those in groups B and C (*P*<0.05). (2) Compared with groups A and E, groups B and C showed increased total gonadotropin (Gn) and days of Gn stimulation (*P*<0.05), and the E_2_ level on trigger day and number of oocytes obtained in group B was significantly less than that in group E (*P*<0.05 or 0.01). Embryo implantation rate, clinical pregnancy rate and live birth rate were increased and miscarriage rate was decreased in groups A, D and E compared with group B (*P*<0.05 or 0.01). (3) There were significant differences among the control group and PCOS groups in some genes that are involved in neuronal-reproductive-metabolic endocrine, transcriptional regulation, cell proliferation and differentiation, etc (*P*<0.05). RNA-Seq results were validated by real time PCR analysis for the expression of follicle stimulating hormone receptor (FSHR), drosophila mothers against decapentaplegic protein 7(Smad7) and glutathione peroxidase 3(GPX3) genes that are known to have an important role in follicular development. Functional alterations were confirmed by the improvement in the ovarian responsiveness to Gn and embryo quality.

**Conclusion:**

Weight loss more than 5kg may regulate the neuroreproductive endocrine hormone secretion, insulin resistance and gene expression profiles of ovarian granulosa cells, so as to improve the ovarian responsiveness to Gn, the embryo quality, embryo implantation rate, clinical pregnancy rate, live birth rate, and reduce the spontaneous abortion rate in obese infertile PCOS patients undergoing IVF-ET.

**Clinical trial registration:**

www.chictr.org.cn, identifier ChiCTR1800018298.

## 1 Introduction

Polycystic ovary syndrome (PCOS) is characterized by polycystic ovaries, ovulatory dysfunction, insulin resistance, hyperandrogenemia or hyperandrogenic changes. The main clinical manifestations are menstrual irregularities, hirsutism or acne, obesity, and infertility. PCOS infertility account for about 70% or more of anovulatory infertility (1). Obesity is an important clinical manifestation of PCOS, and some studies have shown that body mass index (BMI) is an independent risk factor for the development of PCOS (2). Studies have shown that obesity is an important risk factor for the development of adverse pregnancy outcomes in PCOS (3), and high BMI can affect oocyte development, fertilization rate and embryo quality, leading to pregnancy failure (4). A study by Giorgio et al (5) showed that weight loss through a controlled low-calorie diet improved BMI in obese PCOS patients, reduced ovarian volume and microfollicular count, and restored the ovulatory cycle, thereby increasing the natural pregnancy rate. The mechanisms by which weight loss improves pregnancy outcomes in obese PCOS patients are not well understood. In addition, an appropriate and effective amount of weight loss needs to be determined.

A study showed that losing 10 to 15 kg on a diet can successfully reverse type 2 diabetes (6). Considering that both PCOS and diabetes are metabolic and endocrine diseases, we hypothesize that weight loss up to a certain number may also improve endocrine metabolism and clinical pregnancy outcomes in obese infertile PCOS patients undergoing IVF-ET. In this study, we will assess whether effective weight management could improve clinical pregnancy outcomes and neuronal-reproductive-metabolic hormones in obese patients with PCOS infertility. The mRNA profiles of follicular granulosa cells from patients will be analyzed using high-throughput sequencing technology since follicular granulosa cells directly influence ovarian functions and are closely related to oocytes quality (7).

## 2 Materials and methods

The study was approved by the ethics committee of the First Hospital of the University of Science and Technology of China (Anhui Provincial Hospital) and all patients volunteered to participate in the study.

### 2.1 Patients

Weight management follow-up was performed in PCOS infertile patients attending the fertility center of the First Hospital of the University of Science and Technology of China (Anhui Provincial Hospital) from September 2016 to September 2021, and 352 obese PCOS infertile patients were followed up with a 6-month pre-treatment of weight loss before IVF-ET (8). And 75 infertile patients undergoing IVF-ET because of tubal infertility were entered into the control group (Group A) if they satisfied the following criteria: undergoing whole embryo freezing for prevention ovarian hyperstimulation syndrome (OHSS); 18.5≤BMI<23.0kg/m^2^; regular menstrual cycles; normal sonographic ovarian appearance; no diabetes or clinical signs of hyperandrogenism.

PCOS infertile patients were divided into four groups according to their weight loss: 0kg (105 patients, group B, represents a weight loss of less than 1kg considering that normal people have about 1kg fluctuation in weight in 24 hours), 1-5kg (98 patients, group C), 5-10kg (87patients, group D) and >10kg (62patients, group E). Inclusion criteria: (1) age between 22-35 years old; (2) those who met the diagnostic criteria for PCOS and infertility diagnosis; (3) those who could not have used medication known to affect reproductive hormones within 3 months prior to enrollment; (4) the male partner had normal semen or mild oligospermia or hypospermia; (5) undergoing whole embryo freezing for prevention ovarian hyperstimulation syndrome (OHSS); (6) the patients voluntarily participated in this study and actively cooperated with the follow-up and clinical observation.

Exclusion criteria for all groups: Age>35 years, follicle stimulating hormone(FSH) >10IU/L, number of antral follicle count(AFC)<5-7 follicles (9, 10), adenomyosis, chocolate cyst of ovary, uterine malformations or adhesions, chromosomal abnormalities, patients undergoing preimplantation genetic testing (PGT) for genetic reasons (11),etc.

#### 2.1.1 Infertility diagnostic criteria

According to the latest definition by the WHO, infertility is defined as a disease characterized by the failure to establish a clinical pregnancy after 12 months of regular, unprotected sexual intercourse or due to an impairment of a person’s capacity to reproduce, either as an individual or with his/her partner (12).

#### 2.1.2 PCOS diagnostic criteria:

According to the 2003 revised European Society for Human Reproduction and Embryology and American Society for Reproductive Medicine (ESHRE/ASRM) expert meeting (Rotterdam meeting) (13). Need two of the following:

①Oligo-ovulation or anovulation; ②Clinical or biochemical signs of hyperandrogenism; ③ Polycystic ovaries on ultrasound.

And exclusion of other causes (Cushing’s syndrome, congenital adrenal cortex, androgen-secreting tumors, etc.).

#### 2.1.3 Diagnositic criteria of obesity:

According to the WHO standard of obesity for Asian population (14)- (15): normal weight (18.5≤BMI<23.0kg/m^2^), overweight (23.0≤BMI<25.0kg/m^2^), obese (BMI≥25kg/m^2^).

### 2.2 Methods

#### 2.2.1 Ovarian stimulation protocol:

All the collected cases were treated with the antagonist protocol which is commonly used in PCOS patients. Serum hormones and vaginal ultrasound were measured at day 2-3 of menstruation to assess the basal ovarian status, and superovulation induction was started when conditions were suitable. Vaginal ultrasound and sex hormones were monitored during controlled ovarian hyperstimulation, and GnRH antagonist was added when follicle diameter grew to 12 mm or more, and continued until the trigger day. When there were more than 2 dominant follicles with diameter of 18mm or more, hCG (Guangdong Lizhu Group Lippo Biochemicals Co., Ltd.) 8000U or Azer (Gonapeptyl, Ferring Pharmaceuticals) 250ug was routinely injected that night to induce the final maturation of follicles in patients without high risk of OHSS. An appropriate smaller dose of h CG (2000IU or 4000IU) plus with 0.2mg triptorelin (Gonapeptyl, Ferring Pharmaceuticals) will be used in patients with a high risk of OHSS (defined as≥25 follicles ≥11 mm in diameter on the day of trigger) (16). Oocytes were retrieved under ultrasound monitoring after 34-36h.

After *in vitro* fertilization, blastocysts were frozen on day 5-6 of culture. Endometrial preparation was performed in an artificial cycle prior to frozen–thawed embryo transfers in the 2nd menstrual cycle after oocytesr etrieval. Patients were advised to maintain the previous lifestyle and diet in order to maintain weight during the interval between the frozen embryo transfer cycle and the egg retrieval cycle. Endometrial preparation for frozen embryo transfer will be started when the patients maintained their weight loss.

#### 2.2.2 Preparation of the endometrium:

Hormone replacement therapy with exogenous estrogen was started at day 2-3 of the menstrual cycle for endometrial preparation. Patients received twice daily 2-3 mg Estradiol Valerate (Bayer) in which the dose was adjusted according to the endometrial thickness measurements. Progesterone was commenced when the endometrium had a trilaminar appearance and the endometrial thickness was ≥7mm (17). One highest quality blastocyst was transferred 5 days after progesterone treatment. Serum hCG was used to determine a pregnancy 12 days after ET.

Blastocyst scoring: The resulting blastocysts are rated according to the Garnder blastocyst grading method on days 5 to 6 of embryo culture (18). According to the blastocyst cavity stage, inner cell mass (ICM) and trophectodenn (TE) score, the blastocyst cavity stage 3 and above, ICM score B or above is regarded as usable blastocyst (≧3BC) and frozen. Blastocysts with a score of ≧ 3BB are high-quality blastocysts. The remaining available embryos are non-quality blastocysts.

A clinical pregnancy was defined as the presence of a gestational sac with fetal heart activity on ultrasound examination on the 28th day of embryo transfer.

#### 2.2.3 Detected indicators:

(1) The age, BMI (weight/height^2,^ expressed as kg/m^2^), Years of infertility, AMH, HOMA-IR([glucose] (mmol/l) × [insulin] (µU/ml)/22.5) (19), FSH, LH, T and E_2_ were analyzed.

(2) Outcome measures: The primary outcome parameter was live birth rate (the number of cyles with live birth/the number of transfer cycles). The main secondary measures included ovarian stimulation characteristics (including the starting dose of Gn, total dose of Gn, the total days of Gn stimulation, E2 level on trigger day and number of oocytes retrieved), fertilization rate(the number of fertilizations/the number of oocytes retrieved), high quality embryo rate(the number of high quality embryos/the number of total embryos), embryo implantation rate(the number of gestational sacs/the number of embryos transferred), clinical pregnancy rate(the number of cycles with clinical pregnancies/the number of transfer cycles), early abortion rate (the number of abortions in first trimester/the number of clinical pregnancies).

#### 2.2.4 Granulosa cells gene expression analysis:

Follicular granulosa cells of six patients in each group were collected randomly with the random number table method. Each assay sample contains granulosa cells from two patients. On the day of oocytes retrieval, follicular fluid was retained after oocytes retrieval, granulosa cells in the follicular fluid were isolated, total RNA of granulosa cells was extracted and quality control was performed, the extracted RNA was reverse transcribed and amplified, cDNA libraries for sequencing were obtained, mRNA sequencing was completed, sequencing data were analyzed and counted, and *P* value and fold change (FC) were used as the basis for screening for differential genes. *P* value and fold change (FC) were used as the basis for screening differential genes, and *P* < 0.05 was considered statistically significant. The differentially expressed genes obtained were tested, functionally analyzed, and the 3 genes (FSHR, Smad7 and GPX3) with obvious differences were selected for qRT-PCR validation experiments, and the primer sequences are shown in **Table 1**.

**Table 1:** Primers Tested for RT-PCR of mRNA.

Name	Forward primer(5'-3')	Reverse primer(5'-3')
FSHR	CTCACCAAGCTTCGAGTCATCCAA;	AAGGTTGGAGAACACATCTGCCTCT.
Smad7	AGCAAGTCAGCACTGCCAAG	TGACAACTGAAATGCTGATCCAAAG
GPX3	GGCTTTGTCCCTAATTTCCAG	AAAGTTCCAGCGGATGTCGT

#### 2.2.5 RNA sample quality test results:

RNA was analyzed and quantified by spectrophotometer (Nanodrop), and A260/A280 was between 1.80 and 2.0, while RIN value > 7.0 was qualified.

#### 2.2.6 High-throughput RNA-sequencing data analysis of differential genes:

The expression of genes was calculated by RPKM, and the raw data were processed by standard to obtain FC. The screening criteria for up-regulated differential genes were: FC≥2, P<0.05; the screening criteria for down-regulated genes were: FC ≤ 0.5, P<0.05. Then the screened differential genes were subjected to GO analysis and KEGG biological pathway analysis.

### 2.3 Statistical analysis

All data were analysed using the Statistical Package for the Social Sciences for Windows (SPSS 24.0 software IBM, NY, USA). The mean ± standard deviation (x ± s) was used to express the measurement data, and the count data was expressed as the rate (%). Rates were compared by the chi-square test or Fisher’s exact test, ANOVA was used for comparison of means between multiple groups, SNK was used for comparison between groups, and *P* < 0.05 indicated that the difference was statistically significant.

## 3 Results

### 3.1 Patients’ characteristic

Relevant patient parameters are presented in **Table 2**. No differences were observed in terms of age and years of infertility (*P*>0.05), AMH were significantly higher in group B, C, D and E compared to group A (*P*<0.001). No significant differences were found in the level of AMH among group B, C, D and E(*P*>0.05).

**Table 2:** Patients’ characteristic.

Group	Number of cases (n)	Age (years)	Years of infertility (years)	AMH (ng/ml)
Group A	75	28.34 ± 4.06	3.45 ± 2.92	3.56 ± 0.86
Group B	105	29.32 ± 4.15	3.85 ± 2.65	10.78 ± 1.59***
Group C	98	28.85 ± 4.20	3.92 ± 2.16	10.23 ± 1.53***
Group D	87	28.65 ± 4.12	3.96 ± 2.48	9.18 ± 1.46***
Group E	62	27.86 ± 3.84	3.69 ± 2.56	9.27 ± 1.32***

***P<0.001 vs. group A.

### 3.2 BMI and HOMA-IR

BMI and HOMA-IR were significantly higher in four PCOS groups (group B, C, D and E) compared to group A (*P*<0.05), and there were no statistically significant differences among the four PCOS groups (*P*>0.05) before weight loss. After weight loss, BMI in group B was significantly higher than that in group A (*P*<0.05), BMI in the group E was significantly lower than that in group B (*P*<0.05), and BMI in the group E was significantly lower than that before weight loss (*P*<0.05). HOMA-IR in group B, C, D and E were significantly higher than that in group A (*P*<0.05), HOMA-IR in the group E was significantly lower than that in group B(*P*<0.05), and the group E was significantly lower than that before weight loss(*P*<0.05). No significant differences were found in the level of FSH among the five groups (*P*>0.05) (**Table 3**).

**Table 3:** BMI and HOMA-IR.

Group	BMI (kg/m^2^ )		HOMA-IR	
	before weight loss	after weight loss	before weight loss	after weight loss
Group A	22.28 ± 1.68	22.08 ± 1.58	1.68 ± 0.87	1.63 ± 0.92
Group B	26.86 ± 2.34*	26.78 ± 2.25*	4.78 ± 1.86*	4.76 ± 1.85**
Group C	26.98 ± 2.16*	25.78 ± 2.12	4.83 ± 1.79*	4.16 ± 1.78*
Group D	27.24 ± 1.23*	24.61 ± 1.18	4.89 ± 1.78*	3.52 ± 1.76*
Group E	27.65 ± 1.15*	22.06 ± 1.22^#@^	4.92 ± 1.82*	2.78 ± 1.89*^#@^

*P<0.05, **P<0.01 vs. group A; ^#^P<0.05 vs. group B; ^@^P<0.05 vs. the group before weight loss.

### 3.3 Basic reproductive hormones

Before weight loss, LH and T in group B, C, D and E were significantly higher than that in group A (*P*<0.05), and there were no statistically significant differences among the four PCOS groups(*P*>0.05). After weight loss, the levels of LH and T in group B, C, D, E were still higher than that in group A (*P*<0.05 or 0.01).The levels of LH and T in group E were significantly lower than that in group B and C (*P*<0.05), and the levels of LH and T in the group E was significantly lower than that before weight loss(*P*<0.05). No significant differences were found in the level of FSH and E_2_ among the five groups (*P*>0.05) (**Table 4**).

**Table 4:** Basic reproductive hormones.

Group	FSH(mIU/L)		LH(mIU/L)		T(nmol/l)		E_2_(pg/ml)	
	before weight loss	after weight loss	before weight loss	after weight loss	before weight loss	after weight loss	before weight loss	after weight loss
Group A	4.21 ± 0.68	4.18 ± 0.71	4.16 ± 1.16	4.18 ± 1.08	1.36 ± 0.45	1.34 ± 0.53	49.23 ± 18.11	49.68 ± 16.12
Group B	5.86 ± 1.19	5.82 ± 1.18	11.18 ± 4.52**	11.12 ± 4.53**	2.73 ± 0.91**	2.72 ± 0.89**	48.58 ± 16.82	48.22 ± 15.98
Group C	5.87 ± 1.15	5.89 ± 1.19	10.34 ± 4.53*	10.02 ± 4.48*	2.54 ± 0.86*	2.35 ± 0.91*	47.34 ± 17.26	47.56 ± 18.32
Group D	5.90 ± 1.21	5.95 ± 1.22	10.25 ± 4.68*	8.25 ± 4.65*	2.51 ± 0.92*	2.08 ± 0.92*	46.96 ± 16.56	46.62 ± 17.36
Group E	5.97 ± 1.23	6.05 ± 1.24	10.12 ± 4.12*	6.12 ± 4.22*^#&@^	2.48 ± 0.76*	1.76 ± 0.74*^#&@^	47.54 ± 16.32	48.28 ± 19.34

*P<0.05, **P<0.01 vs. group A; ^#^P<0.05 vs. group B; ^&^P<0.05 vs. Group C; ^@^P<0.05 vs. the group before weight loss.

### 3.4 Stimulation cycle characteristics

The starting dose of Gn in group B was significantly higher than group A (*P*<0.05), and the starting dose of Gn in group D and E was significantly lower than group B(*P*<0.05). The total dose of Gn in group B and C were significantly higher than group A (*P*<0.05), and the total dose of Gn in group E was lower than that in group B and C (*P*<0.05). The Days of Gn stimulation in group B was significantly higher than that in group A and E (*P<0*.05),there were no significant differences in the Days of Gn stimulation among group A,C, D and E (*P*>0.05). There are no significant differences in the starting dose of Gn, the total dose of Gn and the days of Gn stimulation between group A and E(*P*>0.05). The E2 level on trigger day and number of oocytes obtained in group D and E were significantly more than that in group A (*P<0*.05 or 0.01), and the E2 level on trigger day and number of oocytes obtained in group E was significantly more than that in group B (*P<0*.05). There were no significant differences in the E2 level on trigger trigger day and number of oocytes obtained among group A, B and C (*P*>0.05) (**Table 5**).

**Table 5:** Stimulation cycle characteristics.

Group	The starting dose of Gn(U)	The total dose of Gn (U)	*Days* *of* *Gnstimulation* (d)	E2 level on trigger day(pmol/l)	Number of oocytes obtained (n)
Group A	150.52 ± 16.48	1378.23 ± 702.56	8.75 ± 1.34	2825.5 ± 1932.5	10.56 ± 2.64
Group B	196.56 ± 24.25*	2231.02 ± 726.56*	12.03 ± 2.26*	3035.2 ± 1936.8	11.76 ± 7.12
Group C	175.20 ± 21.50	2026.34 ± 732.21*	11.89 ± 2.05	3279.9 ± 1867.6	12.26 ± 7.23
Group D	155.45 ± 23.26^#^	1723.25 ± 721.16	10.24 ± 2.02	3768.6 ± 1923.7*	15.45 ± 7.21*
Group E	154.50 ± 22.55^#^	1536.23 ± 714.28^#&^	9.86 ± 1.98^#^	3996.5 ± 1867.6**^#^	16.64 ± 7.28**^#^

*P<0.05, **P<0.01 vs. group A; ^#^P<0.05 vs. group B; ^&^P<0.05 vs. Group C.

### 3.5 Embryology outcome

The rate of high-quality embryos in group B was significantly lower than that in group A (*P*<0.05). The rate of high-quality embryos in group E was significantly Higher than that in group B(*P*<0.05). There were no significant differences in the rate of high-quality embryos between group C, D and E (*P*>0.05). There was no significant differences in the rate of high-quality embryos between A and E (*P*>0.05). No significant differences were found in the fertilization rate among the five groups (*P*>0.05) (**Table 6**).

**Table 6:** Embryology outcome.

Group	Fertilization rate [%(n/N)]	High quality embryo rate [% (n/N)
Group A	71.50 (564/789)	45.01 (248/551)
Group B	68.02 (838/1232)	36.32 (296/815)*
Group C	69.02 (829/1201)	39.90 (322/807)
Group D	69.94 (940/1344)	41.09 (376/915)
Group E	70.00 (721/1030)	43.11 (303/703)^#^

*P<0.05 vs. group A; ^#^P<0.05 vs. group B.

### 3.6 Pregnancy outcome

No significant differences were found in the rate of high-quality embryos transferred among the five groups (*P*>0.05). The embryo implantation rates, clinical pregnancy rate and live birth rates in group B and C were significantly lower than group A(*P*<0.05 or 0.01), and the embryo implantation rates, clinical pregnancy rate and live birth rates in group D and E were significantly higher than that in group B(*P*<0.05). The early abortion rates in group B and C were significantly higher than that in group A (*P*<0.05 or 0.01), and the early abortion rates in group D and E were significantly lower than that in group B(*P*<0.05). No significant differences were found in the pregnancy outcome among the group C,D and E(*P*>0.05). No significant differences were found in the the pregnancy outcomes between the group A and E (*P*>0.05) (**Table 7**).

**Table 7:** Pregnancy outcome.

Group	Rate of high-quality embryos transferred (%, n)	Embryo implantation rate (%, n)	Clinical pregnancy rate (%, n)	Early abortion rate (%, n)	Live birth rate(%, n)
Group A	76 (57/75)	65.33 (49/75)	64 (48/75)	10.42 (5/48)	58.67 (44/75)
Group B	71.40 (75/105)	42.82 (45/105)**	40.95 (43/105)**	32.56 (14/43)**	29.5 (31/105)**
Group C	73.47 (72/98)	51.02 (50/98)*	48.98 (48/98)*	22.92 (11/48)*	39.81 (39/98)*
Group D	74.71 (65/87)	64.37 (56/87)**^#^**	63.22 (55/87)**^#^**	12.73 (7/55)**^#^**	56.32 (49/87)**^#^**
Group E	75.81 (47/62)	62.90 (39/62)**^#^**	61.29 (38/62)**^#^**	13.16 (5/38)**^#^**	54.83(34/62)**^#^**

*P<0.05, **P<0.01 vs. group A; ^#^P<0.05 vs. group B.

### 3.7 RNA high-throughput sequencing data analysis

#### 3.7.1 Analysis of sample gene expression differences

This experiment was performed using DESeq2 R software (1.16.1) for differential expression analysis between two comparative combinations (two biological replicates per group). DESeq2 provides statistical procedures for identifying differentially expressed genes using a model based on a negative binomial distribution. P-values were adjusted using the method of Hochberg and Benjamini and were used primarily to control the false discovery rate of the experimental data. Genes with adjusted P values <0.05 were found to be assigned as differentially expressed by DESeq2. Differential expression analysis for both conditions was performed using the edgeR R software package (3.18.1). There were 337 up-regulated expressed genes and 367 down-regulated expressed genes in the weight loss 1-5 kg group compared to the weight loss 0 kg group; 485 up-regulated expressed genes and 315 down-regulated expressed genes in the weight loss 5-10 kg group compared to the weight loss 0 kg group; and 1056 up-regulated expressed genes and 654 down-regulated genes in the weight loss >10 kg compared to the weight loss 0 kg group (**Table 8**).

**Table 8:** Statistics of differential genes.

Experimental group	Control group	Up-regulation of expressed genes	Down-regulation of expressed genes	Differentially expressed genes	DESeq2 pvalue
Weight loss 0kg	Control	691	782	1473	*<0.05*
Weight loss 1-5kg	Control	737	688	1425	*<0.05*
Weight loss 5-10kg	Control	628	430	1058	*<0.05*
Weight loss >10kg	Control	858	509	1367	*<0.05*
Weight loss 1-5kg	Weight loss 0kg	337	367	704	*<0.05*
Weight loss 5-10kg	Weight loss 0kg	485	315	800	*<0.05*
Weight loss >10kg	Weight loss 0kg	1056	654	402	*<0.05*
Weight loss 5-10kg	Weight loss 1-5kg	365	203	568	*<0.05*
Weight loss >10kg	Weight loss 1-5kg	1672	637	1035	*<0.05*
Weight loss >10kg	Weight loss 5-10kg	536	264	272	*<0.05*

#### 3.7.2 Co-expressed differential genes

**Figure 1:**
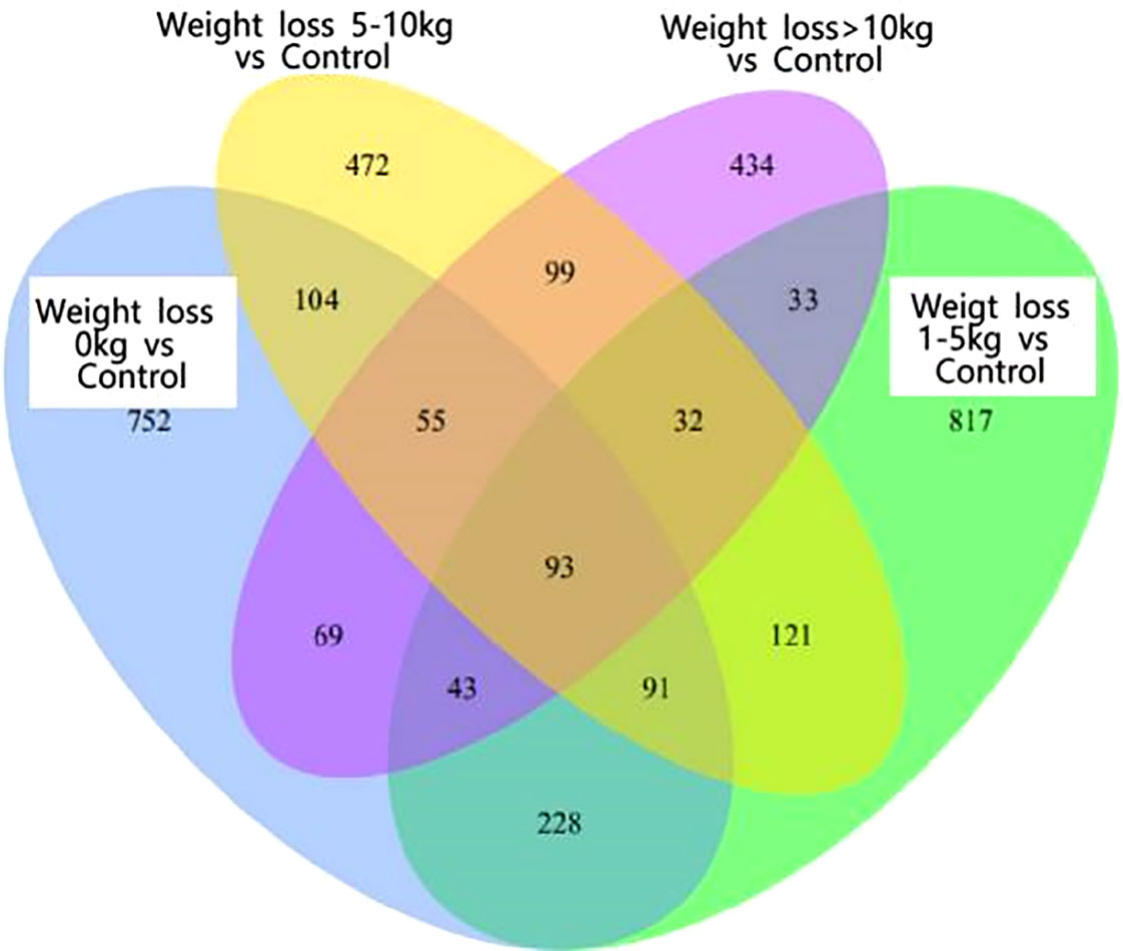
Venn1and.

**Figure 2:**
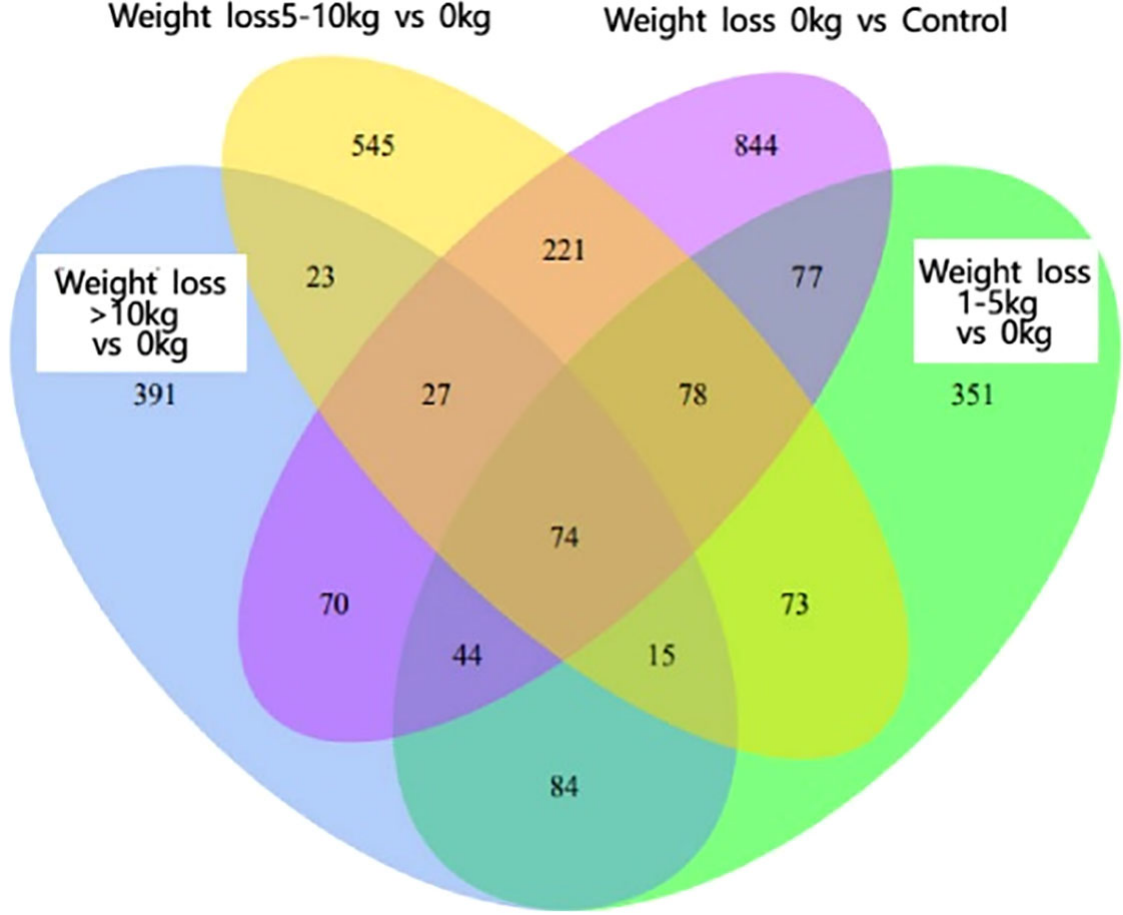
Venn2 Co-expressed differential genes.

As shown in **Figure 1**, there were 93 co-expressed differential genes in the four groups of patients with weight loss 0 kg, weight loss 1-5 kg, weight loss 5-10 kg, and weight loss >10 kg compared with the Control group, indicating that there were at least 93 differential genes in this sample when PCOS patients were compared with normal control patients. As shown in **Figure 2**, the weight loss 1-5 kg, weight loss 5-10 kg, and weight loss >10 kg groups shared 74 co-expressed differential genes compared with the weight loss 0 kg group, indicating that the differentially expressed genes in PCOS patients after weight loss were at least 74 co-expressed differential genes.

#### 3.7.3 Differential gene clustering results

**Figure 3:**
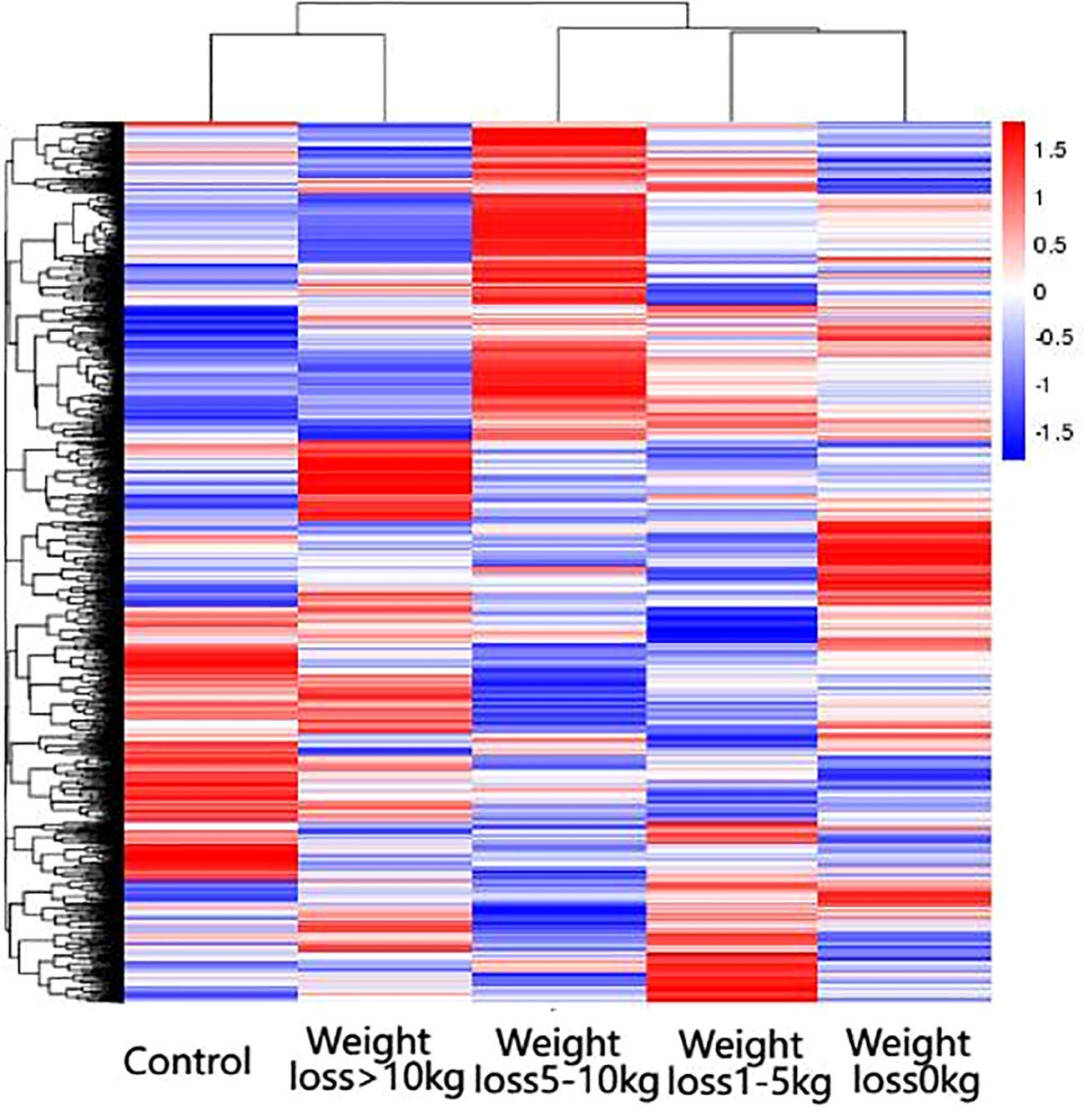
Results of differential gene clustering. Horizontal coordinates are groups, vertical coordinates are normalized values of differential gene FPKM, horizontal comparison, expression of the same gene in samples from different groups, red represents high expression, blue represents low expression.

The differential genes of the five groups of samples were analyzed by clustering, which means that the genes with more similar expression in each group of samples were clustered together, indicating that they might be involved in some metabolic pathways and signaling pathways together or might have a common function. As shown in **Figure 3**, the gene expression of the weight loss 1-5kg group was mostly similar to that of the weight loss 0kg group, and the gene expression of the weight loss>10 kg group was mostly similar to that of the Control group. The results show that the alteration of gene expression in patients with weight loss less than 5kg is not obvious, and the gene expression in patients with weight loss more than 10kg tends to normal control group patients (**Figure 3**).

#### 3.7.4 GO functional enrichment analysis of differentially expressed genes

**Figure 4:**
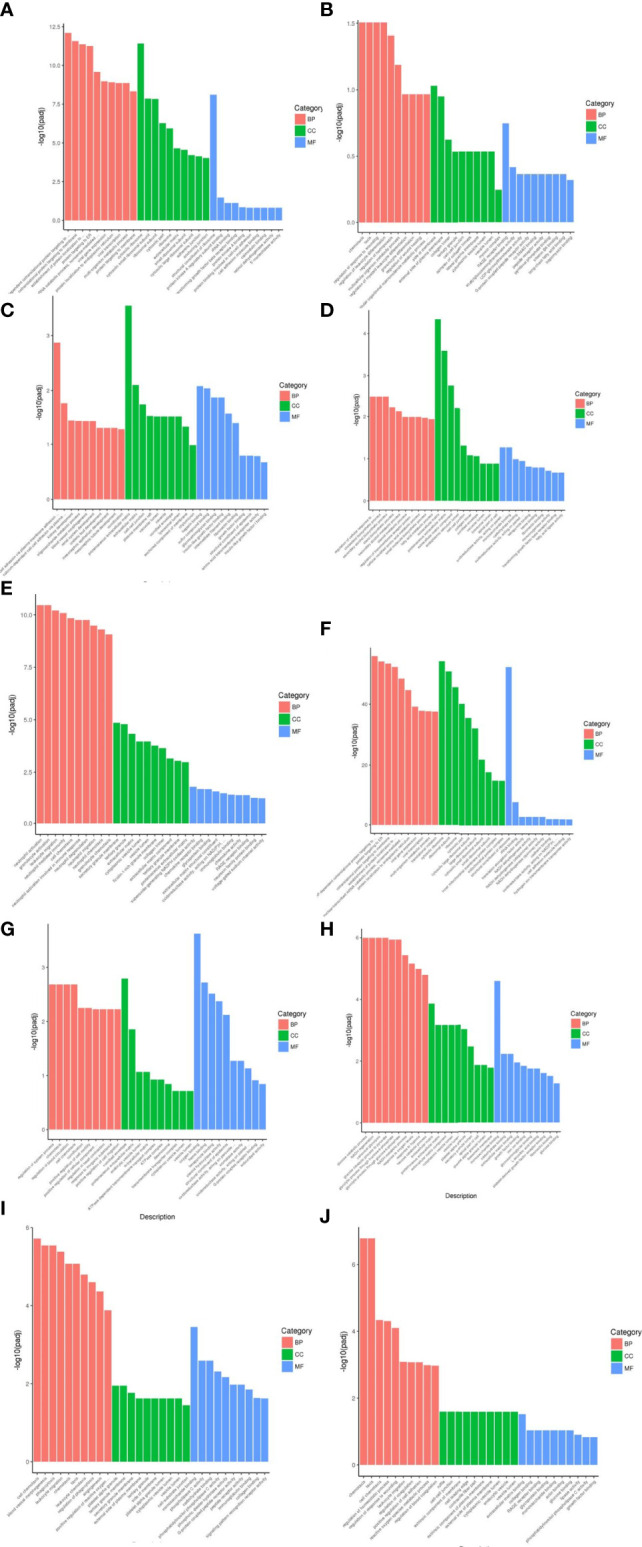
Results of GO functional enrichment analysis of differentially expressed genes. From the results of GO enrichment analysis, the most significant 30 Term were selected to draw bar charts for display, and if less than 30, all Term were plotted, with bar charts drawn by the three major categories of biological process, cellular component and molecular function and up- and down-regulation of differential genes. BP denotes biological process, CC denotes cellular component, MF denotes molecular function. **(A)** Weight loss 1-5kg vs Control, **(B)** Weight loss 1-5kg vs Weight loss 0kg, **(C)** Weight loss 5-10kg vs Weight loss 1-5kg, **(D)** Weight loss 5-10kg vs Control, **(E)** Weight loss 5-10kg vs Weight loss 0kg, **(F)** Weight loss >10kg vs Weight loss 1-5kg, **(G)** Weight loss >10kg vs Weight loss 5-10kg, **(H)** Weight loss >10kg vs Control, **(I)** Weight loss >10kg vs Weight loss 0kg, **(J)** Weight loss 0kg vs Control.

From the results of GO enrichment analysis, the most significant 30 Term were selected for bar graph display according to three major categories of biological processes, cellular components and molecular functions, mainly involved in multiple branching responses such as involvement in cytokine activity, cell differentiation and proliferation, immunophosphorylation, cellular autophagy and transcriptional regulation, and inflammatory response (**Figure 4**).

#### 3.7.5 KEGG pathway enrichment analysis of differentially expressed genes

**Figure 5:**
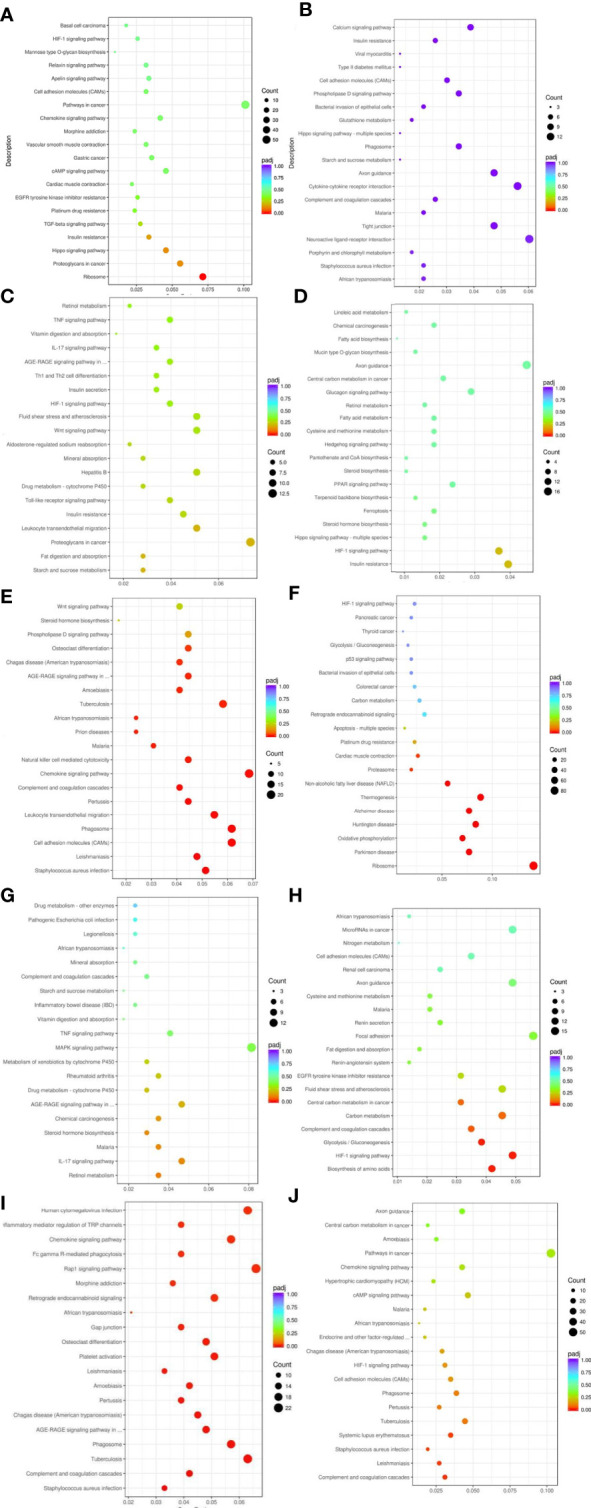
Results of KEGG pathway enrichment analysis of differentially expressed genes. From the KEGG enrichment results, the 20 most significant KEGG pathways were selected to plot scatter plots for display, and if there were less than 20, all pathways were plotted, as shown below. The horizontal coordinate of the graph is the ratio of the number of differential genes annotated to the total number of differential genes on the KEGG pathway, the vertical coordinate is the KEGG pathway, the size of the dot represents the number of genes annotated to the KEGG pathway, and the colour from red to purple represents the significance level of the enrichment. **(A)** Weight loss 1-5kg vs Control, **(B)** Weight loss 1-5kg vs Weight loss 0kg, **(C)** Weight loss 5-10kg vs Weight loss 1-5kg, **(D)** Weight loss 5-10kg vs Control, **(E)** Weight loss 5-10kg vs Weight loss 0kg, **(F)** Weight loss >10kg vs Weight loss 1-5kg, **(G)** Weight loss >10kg vs Weight loss 5-10kg, **(H)** Weight loss >10kg vs Control, **(I)** Weight loss >10kg vs Weight loss 0kg, **(J)** Weight loss 0kg vs Control.

KEGG pathway analysis of differentially expressed genes in five groups of patients identified multiple signaling pathways, including HIF-1 signaling pathway, regulation of endocrine and other factors, complement system, digestion and absorption of fat, regulation of inflammatory mediators by TRP channels, cAMP signaling pathway, TGF-β signaling pathway, insulin resistance, Hippo signaling pathway, AGE-RAGE signaling pathway, etc (**Figure 5**)

### 3.8 RT-PCR validation analysis

**Figure 6:**
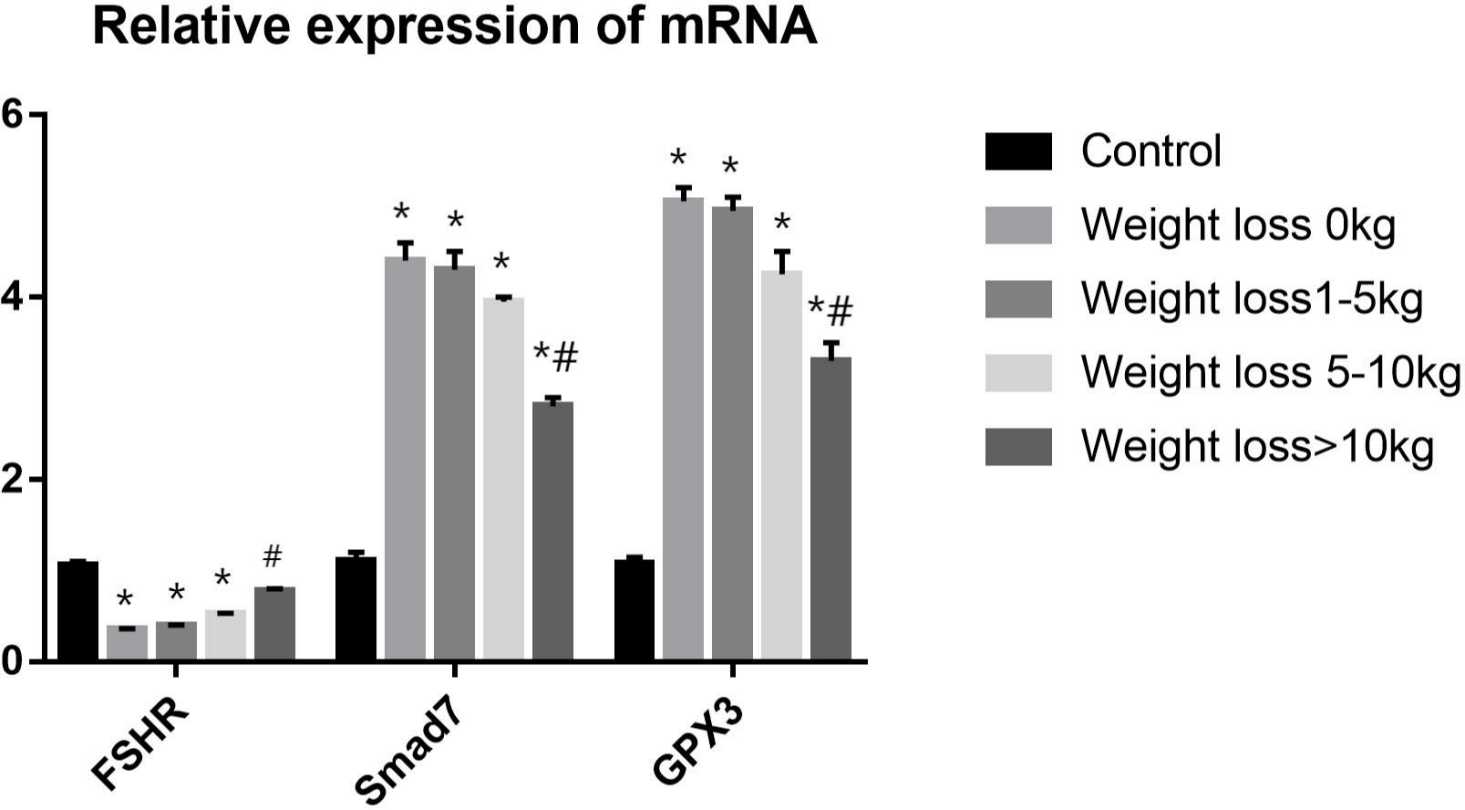
RT-PCR validation analysis.*P<0.05 vs. Control group; ^#^ *P*<0.05 vs. Weight loss 1-5kg group.).

Three differentially expressed genes were selected based on fold change and tested using qRT–PCR for validation: FSHR, Smad7 and GPX3. Analysis of the qRT-PCR data indicated that the genes of FSHR expression was significantly lower in weight loss 0kg,1-5kg and 5-10kg groups as compared to control group(*P*<0.05), and the FSHR expression in the weight loss >10kg group was significantly higher than that in the weight loss 0kg group(*P*<0.05). No statistically significant difference in FSHR expression in the weight loss >10kg group as compared to the control group (*P*>0.05). The Smad7 and GPX3 expressions were significantly higher in PCOS groups as compared to control group A(*P*<0.05), and the Smad7 and GPX3 expressions in the Weight loss >10kg group was significantly lower than that in the weight loss 0kg group(*P*<0.05). No significant differences were found in the Smad7 and GPX3 expressions among the weight loss 0kg,1-5kg and 5-10kg group(*P*>0.05). The results were consistent with the sequencing results (**Figure 6**).

## 4 Discussion

**Figure 7:**
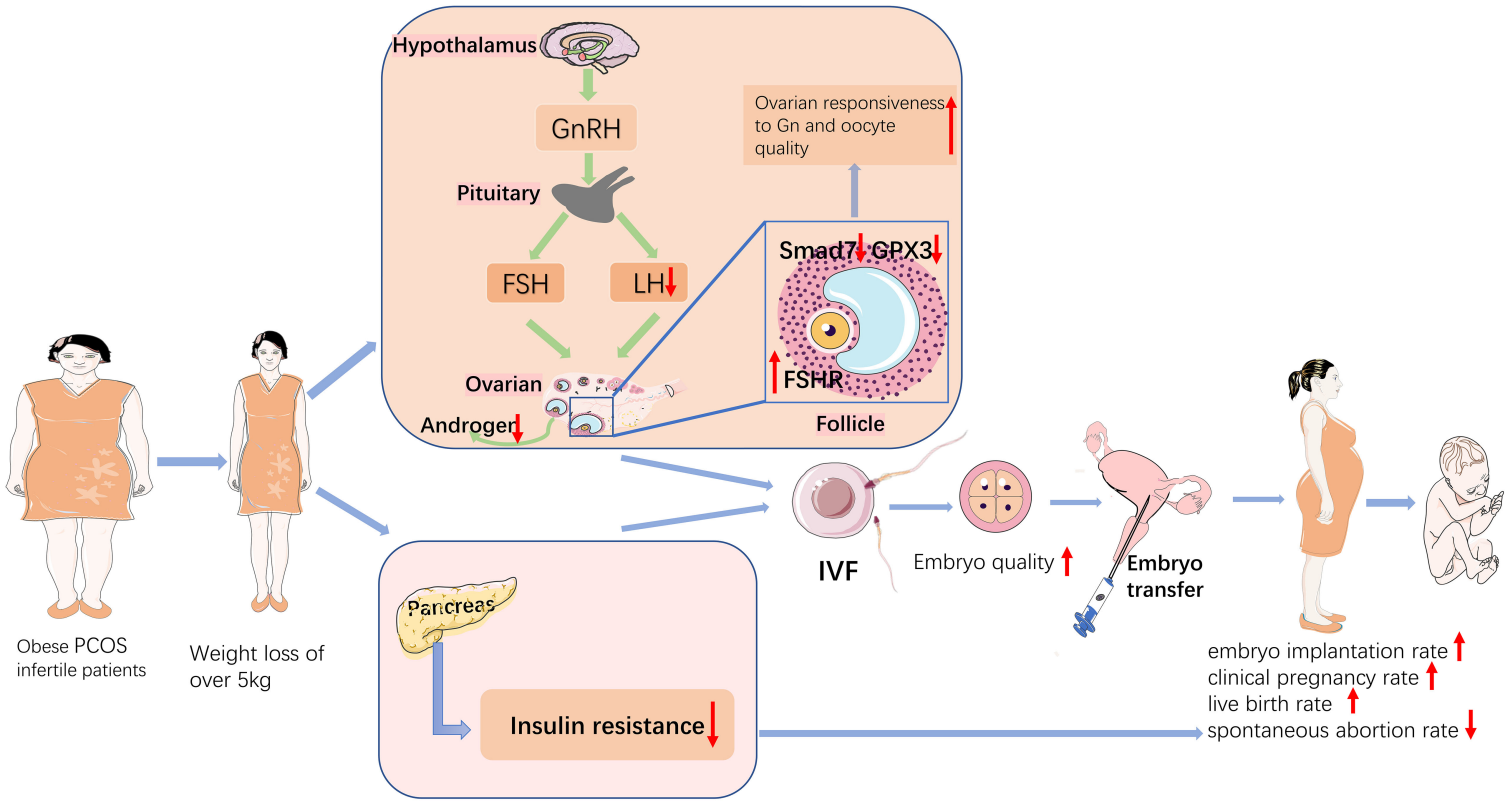
Schematic diagram of the mechanisms that weight loss improves pregnancy outcomes in obese infertile PCOS patients undergoing IVF-ET.

Obesity has become a growing matter of public health concern worldwide, and the mechanisms by which obesity affects reproductive function in patients with PCOS are complex and still not completely understood. A clear relationship exists between obesity, metabolic dysregulation, and ovarian dysfunction (20). Previous studies have shown that either complex endocrine factors or metabolic alterations resulting in overweight or obesity can affect follicular growth, embryonic development, and embryo implantation (21). Weight loss has been used clinically as one of the treatment modalities for PCOS, and this study focused on the effect of weight loss on pregnancy outcome and follicular granulosa cell gene expression in obese PCOS infertility patients undergoing IVF-ET, and analyzed the mechanism of action by studying the differential genes of PCOS patients. In summary, pretreatment with weight loss prior to IVF-ET improved pregnancy outcomes and mRNA profile in obese PCOS infertile patients. This is the first study to analyse the effect of different levels of weight loss on the gene expression profile of follicular granulosa cells in patients with PCOS infertility. The findings of this study are summarized in **Figure 7**.

### 4.1 Clinical outcomes and the effect of weight loss in obese PCOS patients

According to our results, PCOS patients with less weight loss required more Gn and days of Gn stimulation, retrieved less oocytes and less high-quality embryos than patients with more weight loss. Similar to the results of a previous study (22), patients with overweight PCOS may have “(Gn) resistance”, which may increase ovarian hyperstimulation during superovulation because of the longer duration and higher dose of Gn compared to patients with normal weight PCOS. Obesity is an important clinical manifestation in patients with PCOS, and some studies have shown that BMI is an independent risk factor for the development of PCOS (23). The results of this study showed that weight loss over 5 kg significantly increased the embryo implantation rates, clinical pregnancy rate and live birth rates, and significantly decreased the miscarriage rate in infertile patients with PCOS compared to those with weight loss 0 kg. Obesity may have an adverse effect on the outcome of assisted reproductive treatment, with miscarriage rates increasing with BMI in women with obese PCOS treated with IVF/ICSI compared to normal weight women, significantly higher than in infertile patients with normal BMI (24). Moreover, obesity leads to impaired production of hormones related to follicular development as well as metabolites (25), thus reducing oocyte quality. Rittenberg et al. (26) concluded that obesity increases the rate of miscarriage and decreases the rate of live births in IVF. It has also been shown that obese PCOS patients have a greater impact on pregnancy outcomes in assisted reproduction. A study showed that clinical pregnancy rates, embryo transfer rates, and live birth rates were significantly lower in obese PCOS patients compared to women with lean PCOS (27). A retrospective analysis by D Best et al. (28), in which 40 studies were included, showed that weight reduction by means of a low-calorie diet and exercise improved ovulation and pregnancy rates. Clark (4) et al. showed that weight loss increased the clinical pregnancy rate and decreased the miscarriage rate in infertile patients with PCOS. The above findings are more consistent with the results of this study.

### 4.2 Neuronal-reproductive-metabolic hormones and the effect of weight loss in obese PCOS patients

Patients with PCOS have abnormal function in hypothalamic-pituitary-ovarian axis and increased frequency of GnRHa secretory pulses of the hypothalamus, acting on the pituitary gland and causing excessive LH secretion (29). Hyperinsulinemia in obese patients promotes androgen production in the adrenal glands and ovaries, and reduces testosterone metabolism, leading to hyperandrogenemia (30). Consistent with the above research, LH and T increase in obese infertile PCOS patients in our study.

Moreover, we found that the LH, T and HOMA-IR of obese infertile patients with PCOS were improved after weight loss, indicating that weight loss may affect the secretion of LH and T by improving function of neuronal-reproductive-metabolic circuits in patients with PCOS. Obesity and insulin resistance are the manifestations of metabolic syndrome in PCOS. According to human and animal studies concerning the effect of insulin in PCOS development, insulin is considered as a co-effector of gonadotropins. Insulin can stimulate LH secretion directly (31), leading to aberrant reproductive function in PCOS. Hypothalamic POMC neurons express both insulin receptor and leptin receptor, and knock-out of insulin receptor and leptin receptor in POMC neurons induced PCOS phenotype, indicating the insulin and leptin can be powerful regulators of both kisspeptin and POMC neurons, which further promote PCOS development (32). The results of a study (33) showed that improvement of insulin sensitivity increases pregnancy rate in infertile PCOS women.

In addition, one study reported that weight loss in obese PCOS patients resulted in increased expression of insulin receptor substrate 1 in the endothelium and increased expression of glucose transporter protein 1, resulting in improved endothelial function (34).Growing pieces of evidence suggest that activated sympathetic nervous system takes part in PCOS and obesity pathogenesis (35). Furthermore, our findings show that clinical pregnancy rates, live birth rates and miscarriage rates in infertile patients with PCOS are significantly improved after weight loss pretreatment. Thus, weight loss can regulate GnRH neuronal activity and ultimately reproductive function by improving insulin resistance, obesity and other metabolic factors in PCOS patients. The results are more consistent with the findings of Baoying Liao et al (36).

### 4.3 Gene expression of follicular granulosa cells and the effect of weight loss in obese PCOS patients

Gene expression of follicular granulosa cells was analyzed to investigate the mechanisms because granulosa cells are important functional cells within the follicle that regulate the maturation and development of the oocyte through a complex connection mechanism (37). Granulosa cells are closely related to oocyte quality and not only affect the development and maturation of oocytes, but also the differentiation and proliferation of granulosa cells directly influence ovarian-related functions such as development and discharge of oocytes, production and secretion of steroid hormones, formation of corpus luteum (7) and even interfere to some extent with embryonic developmental potential.

In this study, transcriptome sequencing was performed to obtain differential gene expression profiles in ovarian granulosa cells of obese PCOS infertile patients undergoing weight loss and normal control infertile patients, and GO functionalenrichment and KEGG pathway enrichment were used to screen and functionally analyze the differentially expressed genes. The results of this study showed that 337 genes were up-regulated and 367 genes were down-regulated in the weight loss 1-5kg group compared with the weight loss 0kg group; 485 genes were up-regulated and 315 genes were down-regulated in the weight loss 5-10kg group compared with the weight loss 0kg group; 1056 genes were up-regulated and 654 genes were down-regulated in the weight loss >10kg group compared with the weight loss 0kg group. These differentially expressed genes were mainly involved in signaling pathways such as inflammatory response, lipid metabolism and insulin-related. Molecular validation of the bioinformatics screened differential genes was performed using real-time quantitative techniques, and both results were consistent, indicating that the data assembled by our group is better for further analysis.

In a cohort study by Mauricio et al. (38), the expression of genes in the insulin resistance signaling pathway in ovarian granulosa cells of normal weight and obese PCOS patients was evaluated and the results showed that 10 genes were overexpressed in obese PCOS patients compared to normal weight PCOS patients. The overexpressed genes are mainly responsible for oocyte proliferation and differentiation, insulin resistance, apoptosis regulation and early embryonic glucose metabolism during oocyte maturation, suggesting the presence of insulin resistance in the follicular environment even in the absence of clinical symptoms, suggesting that these alterations may be associated with obese women with PCOS with a poorer prognosis for follicular development and oocyte maturation. The GO functional enrichment and KEGG pathway enrichment in our study, showed that differential genes are also mainly responsible for oocyte proliferation and differentiation, insulin resistance, apoptosis regulation and early embryonic glucose metabolism during oocyte maturation etc.

Our study found that the expression of FSHR was decreased, and the expression of Smad7 and GPX3 were significantly increased in follicular granulosa cells of PCOS patients. With the increase of weight loss, the expression of FSHR increased, Smad7 and GPX3 decreased. This indicates that weight loss may improve clinical outcomes by affecting the gene expression in follicular granulosa cells, such as FSHR, Smad7 and GPX3.

The hypothalamus-pituitary-ovary (HPO) axis controls ovarian development and function including follicle recruitment, oocyte selection, regular cycling, and steroid hormone biosynthesis (39). FSH-receptors (FSHR) on granulosa cells in the ovary cells control the recruitment and maturation of gonadal stem cells and the processing and transport of steroid hormones (40). HPO axis imbalance is considered as an important pathophysiology underlying PCOS, indicating that central modulation, especially the abnormal activation of hypothalamic GnRH neurons plays a vital role in PCOS development. The results of this study showed that FSHR expression was low in obese PCOS patients and FSHR expression can be improved through weight loss, which led to improved ovarian Gn responsiveness, reduced Gn dosage during ovulation cycles, and improved follicle quality, and then improved embryo quality.

It has been found (41) that Smad7 acts as a mediator of TGF-β (Transforming growth factor) mediated apoptosis and that Smad7 dysregulation leads to abnormal granulosa cell growth and development, thus affecting embryo quality and increasing the risk of pregnancy failure. Our study suggested that weight loss over 10kg in PCOS infertile patients can significantly reduce the expression of Smad7, thus improving the regulation of TGF-β signaling pathway, which in turn increases follicle quality and improves pregnancy outcome.

It has been shown that GPX3 gene is closely associated with oocyte maturation and embryo quality (42). Regarding the role of GPX3, Xin Huang (43)et al. suggested that GPX3 could be used as an embryo quality monitoring marker and the lower the expression of GPX3, the greater the likelihood of oocytes developing into blastocysts. It suggests that GPX3 can affect embryo quality in PCOS patients. In the present study, the expression of GPX3 was higher in all PCOS samples than in normal controls (gene expression was higher than 2-foldin all groups), and there was no significant difference in the expression of GPX3 among the group weight loss 0kg,1-5kg and 5-10kg. Weight loss of more than 10 kg in infertile patients with PCOS significantly reduces gene expression of GPX3. It is hypothesized that the high expression of the gene GPX3 in PCOS infertile patients affects the development of oocytes into blastocysts, which eventually leads to pregnancy failure, and weight loss in infertile patients with PCOS reduces gene expression of GPX3 and thus improves pregnancy outcomes.

Notably, our study found that a weight loss of more than 5kg improved the clinical performance of infertile patients with PCOS, but only a weight loss of more than 10kg resulted in significant changes in gene expression of follicular granulosa cells. We hypothesize that the mechanisms involved in the effect of weight loss may include improvements in the neuronal-reproductive-metabolic hormones, endocrine environment, and other gene expression in follicular granulosa cells. We need to conduct more in-depth studies in the future.

The shortcoming of this study is that the patients lost weight in various ways, such as taking orlistat or metformin, dieting, and exercising, so it is uncertain whether different ways of weight loss have an effect on the clinical characteristics, pregnancy outcome, and gene expression of PCOS patients. In the subsequent study, we will further investigate the effect of different weight reduction modalities on PCOS. This is a sample of limited size from China in Asia. We look forward to data from more centers, countries and regions.

## Data availability statement

The data presented in the study are deposited in the NCBI repository, accession number PRJNA882424.

## Ethics statement

The studies involving human participants were reviewed and approved by the ethics committee of the First Hospital of the University of Science and Technology of China (Anhui Provincial Hospital). The patients/participants provided their written informed consent to participate in this study.

## Author contributions

LW designed the study and wrote the manuscript. QF processed the data and wrote the manuscript. MW and ZF carried out the cycle monitoring and stimulation regimens. YW, XZ, FL and BX carried out PCR, gene expression assays and data analysis. XT, HH and RJ critically reviewed the final version of the manuscript. All authors contributed to the article and approved the submitted version.

## Funding

Our study was supported by the General project of National Natural Science Foundation of China (No. 81971446, 81373671 and 8167381) and Natural Science Foundation of Anhui Province (No.2208085MH206).

## Conflict of Interest

The authors declare that the research was conducted in the absence of any commercial or financial relationships that could be construed as a potential conflict of interest.

## Publisher’s note

All claims expressed in this article are solely those of the authors and do not necessarily represent those of their affiliated organizations, or those of the publisher, the editors and the reviewers. Any product that may be evaluated in this article, or claim that may be made by its manufacturer, is not guaranteed or endorsed by the publisher.
